# Effect of a pediatric fruit and vegetable prescription program on child dietary patterns, food security, and weight status: a study protocol

**DOI:** 10.1186/s12889-022-12544-y

**Published:** 2022-01-21

**Authors:** Amy Saxe-Custack, David Todem, James C. Anthony, Jean M. Kerver, Jenny LaChance, Mona Hanna-Attisha

**Affiliations:** 1 Division of Public Health, Department of Pediatrics and Human Development, Michigan State University–Hurley Children’s Hospital Pediatric Public Health Initiative, Flint, MI USA; 2 Department of Epidemiology and Biostatistics, College of Human Medicine, Michigan State University, East Lansing, MI USA; 3 Division of Public Health, Michigan State University–Hurley Children’s Hospital Pediatric Public Health Initiative, Flint, MI USA

**Keywords:** Fruit and vegetable prescription, Child, Adolescent, Nutrition

## Abstract

**Background:**

Although nutrients in fruits and vegetables are necessary for proper development and disease prevention, most US children consume fewer servings than recommended. Prescriptions for fruits and vegetables, written by physicians to exchange for fresh produce, address access and affordability challenges while emphasizing the vital role of diet in health promotion and disease prevention. Michigan’s first fruit and vegetable prescription program (FVPP) exclusively for children was introduced in 2016 at one large pediatric clinic in Flint and expanded to a second clinic in 2018. The program provides one $15 prescription for fresh produce to all pediatric patients at every office visit. Prescriptions are redeemable at a year-round farmers’ market or a local mobile market. The current study will assess the impact of this FVPP on diet, food security, and weight status of youth.

**Methods:**

Demographically similar pediatric patient groups with varying levels of exposure to the FVPP at baseline will be compared: high exposure (> 24 months), moderate exposure (12–24 months), and no previous exposure. Data collection will focus on youth ages 8–16 years. A total of 700 caregiver-child dyads (one caregiver and one child per household) will be enrolled in the study, with approximately 200 dyads at clinic 1 (high exposure); 200 dyads at clinic 2 (moderate exposure), and 300 dyads at clinic 3 (no previous exposure). Children with no previous exposure will be introduced to the FVPP, and changes in diet, food security, and weight status will be tracked over two years. Specific aims are to (1) compare baseline diet, food security, and weight status between pediatric patients with varying levels of exposure to the FVPP; (2) measure changes in diet, food security, and weight status before and after never-before-exposed children are introduced to the FVPP; and (3) compare mean 12- and 24-month follow-up measures of diet, food security, and weight status in the initial no exposure group to baseline measures in the high exposure group.

**Discussion:**

Completion of study aims will provide evidence for the effectiveness of pediatric FVPPs and insights regarding the duration and intensity of exposure necessary to influence change.

**Trial registration:**

The study was registered through clinicaltrials.gov [ID: NCT04767282] on February 23, 2021.

## Background

The 2020–2025 Dietary Guidelines for Americans state that children and adolescents should consume fruits and vegetables regularly to support healthy development and prevent chronic disease. The US Department of Agriculture recommends a minimum daily intake of 1.5 cups of fruit and 1.5 cups of vegetables for females ages 9–13 years and 1.5 cups of fruit and 2 cups of vegetables for males ages 9–13 years [1, 2]. These recommendations often are unfulfilled, especially within low-income households [3–8]. While most young people across the US struggle to attain even the minimum daily recommendations related to fruit and vegetable consumption, [7, 8] ultra-processed foods now comprise the majority of total energy intake among US youths 2–19 years [9]. Increased adiposity related to shifts in diet and exercise have prompted public health action to encourage intake of fresh or minimally processed food among children and adolescents [10].

Eating patterns during childhood and adolescence are important to long-term diet and health [11–13] and play a key role in the development of diet-related chronic health conditions [13–19]. To support healthy food choices while also addressing food access challenges, some health care clinics have introduced fruit and vegetable prescription programs (FVPPs) [20–23]. The programs differ in design and scope, yet most include health provider-issued prescriptions that patients can exchange, without charge, for fresh produce at local farmers’ markets, mobile markets, or food stores. While programs are increasingly available across the country, most target adults with chronic health conditions, offering fruit and vegetable prescriptions as a disease-management strategy [22–25]. Few studies have examined whether, and to what degree, exposure to pediatric FVPPs influences the diet and health of youth [26, 27]. With childhood and adolescence representing a critical period of development when many are establishing lifelong dietary behaviors [11–13], these programs are likely to have meaningful health implications.

In February 2016, a large university-affiliated pediatric clinic in a low-income urban community introduced Michigan’s first pediatric FVPP. The program included physician-issued $15 prescriptions for fresh produce that were distributed after the office visit. Prescriptions were redeemable only for fresh fruits and vegetables at the downtown farmers’ market or a local mobile market that traveled throughout the city. Caregivers whose children were exposed to this year-round FVPP viewed it as effective in improving child dietary patterns and household food security [28]. Moreover, preliminary research, following an expansion of the identical prescription program to a second pediatric clinic, disclosed improvements in food security measures and dietary behaviors of children following one year of exposure to this program [26].

In 2018, the US Farm Bill included produce prescription programs, through the Gus Schumacher Nutrition Incentive Program, for the first time [29]. The legislation provided federal grant support for new and existing FVPPs. Still, these programs continue to focus largely on adults with diet-related chronic health conditions. Few programs are designed specifically to promote healthy eating and prevent disease among vulnerable youth. The current study, conducted across three large pediatric clinics in Flint, Michigan, is designed to provide empirical evidence to evaluate the effectiveness of pediatric fruit and vegetable prescriptions provided to youth and gain insights regarding the duration and intensity of exposure needed to influence changes in food security, dietary patterns, and weight status among children and their caregivers.

### Study aims and hypotheses

This study will assess whether exposure to a pediatric FVPP that provides one $15 prescription for fresh produce to every child at each office visit might account for later changes in dietary intake, food security, and weight status. Demographically similar pediatric patient groups with varying levels of exposure to the FVPP at baseline will be compared: high exposure (> 24 months), moderate exposure (12–24 months), and no previous exposure. Children with no previous exposure will be introduced to the FVPP, and researchers will track changes in dietary intake, food security, and weight status over two years. The specific aims of the study are to (1) compare baseline dietary intake, food security, and weight status between pediatric patients with varying levels of exposure to the FVPP; (2) measure changes in diet, food security, and weight status when  never-before-exposed children are introduced to the FVPP; and (3) compare mean 12- and 24-month follow-up measures of dietary intake, food security, and weight status in the initial no exposure group to baseline measures in the high exposure group. A central expectation is that greater exposure to this FVPP will promote intake of fruits and vegetables, better food security, and lower rates of childhood obesity.

The current study, in design and approach, is grounded in the theoretical framework of Bandura’s Social Cognitive Theory (SCT). SCT explains behavior using a three-stage, dynamic model linking personal factors, environmental factors, and behavior [30, 31]. This study’s SCT framework involves collaborations with partnering physicians and local agricultural leaders to support environmental change and social learning. Since children’s dietary intake is generally guided by their caregivers, critically important environmental factors, such as access to fruits and vegetables as well as caregiver modeling of the healthy behaviors, are of particular importance [32, 33]. Consistent with SCT, we also posit that self-efficacy is central to behavior change. Accordingly, self-efficacy for consuming fruits and vegetables, which refers to one’s judgment over one’s capability to enact the behavior, is important to change dietary behavior [34]. By grounding the study with the theoretical framework of SCT, we suggest that pediatrician issuance of a prescription for fruits and vegetables to every child at each office visit will promote food access and self-efficacy to consume healthy foods. Furthermore, pediatrician messaging to children and caregivers will influence health-promoting behaviors linked to increased consumption of fruits and vegetables.

## Methods/Design

### Study Design

This study will include a cross-sectional analysis of data from a consecutive sample of caregiver-child dyads with varying levels of exposure to a (FVPP) that provides one $15 prescription for fresh produce to every child during pediatric office visits. Considering the importance of dietary patterns during childhood on long-term diet and health [11–13] alongside evidence that indicates adolescence is the specific period when food insecurity has the greatest potential to negatively impact the diet [35], data collection and analyses will focus specifically on children and adolescents ages 8–16 years. A total of 700 caregiver-child dyads (one caregiver and one child per household) will be enrolled in the study, with approximately 200 dyads enrolled at clinic 1 (high exposure); 200 dyads enrolled at clinic 2 (moderate exposure), and 300 dyads enrolled at clinic 3 (no previous exposure to the FVPP). A description of the clinics is provided with our explanation of the ‘Setting’ for this study.

Dyad enrollment will be based on exposure to the FVPP as defined by caregiver-reported and clinic-verified length of time that the participating child has been a patient at one of three partnering clinics. Accordingly, we expect dyads to cluster based on pediatric clinics as follows: clinic 1 (high exposure), clinic 2 (moderate exposure), and clinic 3 (no previous exposure). A sample of approximately 200 pediatric patients newly exposed to the FVPP at clinic 3 will be followed for a period of two years as part of a non-controlled longitudinal intervention trial that will examine changes in dietary patterns, food security, and weight status over time.

### Setting

Flint, Michigan, is a low-income urban city where an approximate 60% of children live in poverty, and full-service grocery stores located inside the city are limited [36]. The city lacks resources and nutritional options, like many low-income urban cities in the US. As a result, many children consume insufficient nutrient-dense foods and excessive poor-quality, calorie-dense foods [20]. Local stores in Flint tend to offer lower-quality foods and fewer healthy foods than offered in higher-income neighborhoods [28, 37–39]. Along with long-lasting issues related to food access and affordability, coupled with recovery from a drinking water crisis [40], the SARS-CoV-2 coronavirus pandemic has disproportionately affected Flint and surrounding Genesee County. Confirmed COVID-19 case prevalence has consistently placed the county in the top ranks (#5) among 83 Michigan counties. In ordinary times, with constrained wealth, and many families in poverty, under- or unemployed, Flint faces extraordinary health challenges. The pandemic has compounded these ordinary challenges, with results that include more hungry children and increased food insecurity.

The current study will occur at three clinics in Flint serving demographically similar patient populations.

- Clinic 1 is a university-affiliated pediatric clinic that introduced Michigan’s first pediatric FVPP. The program began as a pilot in February 2016. After several program iterations and refinements, all patients currently receive one $15 prescription for fresh fruits and vegetables at every office visit. Pediatricians at clinic 1 have distributed approximately 40,000 prescriptions since the FVPP was introduced.
- Clinic 2 is one of the largest private-practice pediatric clinics in Flint, serving over 3,000 young patients. The FVPP was introduced at clinic 2 in August 2018 as part of a grant to assess the feasibility and preliminary effectiveness of the FVPP at a second clinic site [26]. Clinic 2 was selected for this expansion because of the clinic size, demographics of the patient population which closely match clinic 1, and use of an electronic medical record (EMR) system that is identical to clinic 1 (allowing easy tracking of monthly prescription distribution rates). Clinic 2 has distributed over 15,000 prescriptions since August 2018.
- Clinic 3 is the largest pediatric clinic in Genesee County, with Flint as its urban center. Expansion of the FVPP to clinic 3 will substantially increase program reach among vulnerable children and families living in Flint. Last year, clinic 3 had over 12,000 pediatric visits. It has a comparable patient population to clinics 1 and 2, serving only children in need who are residents of Genesee County.

### Participants and recruitment

A sample of 250 caregiver-child dyads presenting at clinic 1, 250 caregiver-child dyads at clinic 2, and 350 caregiver-child dyads at clinic 3 will be assessed for eligibility, with 700 total dyads enrolled (anticipated 10–15% not meeting eligibility and 10% refusal based on a previous study). To control for confounders, we will adopt a balancing strategy that consists of recruiting in the study children who have approximately the same age, sex, and race across the three clinical settings. These children will not be balanced according to variables pertaining directly to the exposures (e.g., duration of FVPP exposure).

With target samples of 200, 200, and 300 caregiver-child dyads enrolled in clinic 1—high exposure, clinic 2—moderate exposure, and clinic 3—no previous exposure, respectively, we have well above 90% power to jointly detect modest relative differences in the baseline mean intake of vegetables of 20% from clinic 2 to clinic 3 and 38% from clinic 1 to clinic 3. This calculation assumes an underdispersed Poisson model with a dispersion parameter of 0.82, derived using previously published data on the mean intake of vegetables of 0.72 cup and a standard deviation of 0.77 cup in the settings with no prior FVPP exposure [20]. Likewise, with 300 caregiver-child dyads enrolled in clinic 3, we have well above 90% power to detect a modest temporal improvement in the mean intake of vegetables of 25% over 12 months (0.72 cup at baseline versus 0.90 cup at 12-month follow-up). This calculation assumes a correlated Poisson model for underdispersed count data with timepoint dispersion parameter of 0.82, and a modest temporal correlation of 0.2. Increasing values of the temporal correlation greatly improves the statistical power. Finally, estimation of FVPP effects will specify two-tailed tests with alpha set at the 0.05 level.

Inclusion criteria for all participants include a restriction to children aged between 8 and 16 years at enrollment; the caregiver and child must speak English. An estimated 2% of the Flint population cannot speak English. A legal guardian must be present at enrollment and must sign an informed consent document; each child must assent to participation. Inclusion criteria for clinic 1 (high exposure) will include participation in the FVPP for over 24 months. Inclusion criteria for clinic 2 participants (moderate exposure) will include participation in the FVPP for 12 to 24 months. Inclusion criteria for clinic 3 participants (no exposure) will include no previous exposure to the FVPP. Potential participants at clinic 3 will be excluded if the household includes a sibling who already enrolled in the research study or if there has been movement of the patient from one clinic to another clinic (< 3% of patients).

### Intervention description

Fruit and vegetable prescriptions, analogous to medical prescriptions, are electronically prescribed by pediatricians and given to patients. They are intended to promote a healthy food environment within the home [41]. For our study, eligible vendors comprise the downtown Flint Farmers’ Market (FFM) and Flint Fresh, a mobile market and food hub that offers locally grown, home-delivered, fresh produce boxes. Vendors accept the prescriptions ($15 each) as if they were gift certificates or vouchers for any fresh fruits or vegetables. In partnership with FFM and Flint Fresh, prescription redemption rates are recorded and tracked. Over time, the redemption of prescriptions has been increasing (from under 30% when the program was initiated to its current redemption rate of approximately 50%).

Clinics 1 and 2 have developed an efficient and reliable method to record and track prescription distribution rates. Fruit and vegetable prescriptions are built using the Epic EMR system, to order and print each prescription, and to track individual and aggregate monthly distribution rates. Key personnel on the current study will train pediatricians and staff at clinic 3 regarding EMR procedures to order, print, and track prescriptions through their EMR system, eClinicalWorks (eCW). The study protocol will capture monthly reports that cover topics such as the number of patients seen in the clinic and prescriptions distributed.

To monitor prescription redemption, FFM vendors will collect paper prescriptions redeemed for fresh produce and turn them into the FFM management office at the end of each month for payment. Prescriptions will be collected, sorted, stored, and entered into a database for tracking purposes. A similar method will be used to track and record monthly prescription redemption through Flint Fresh, including tracking of virtual prescriptions, which were introduced during the COVID-19 pandemic as pediatric telehealth visits increased.

### Data collection procedures

Consent/assent procedures and data collection at baseline will be carried out in a private clinic location, such as a patient examination room. A trained research assistant (RA) will follow our study’s Institutional Review Board (IRB)-approved protocol for consent, child assent, and data collection (e.g., of dietary data). The RA will provide detailed instructions regarding the study, survey completion, and how to make use of an iPad and its interface with a secure digital platform (Research Electronic Data Capture, or REDCap).

Each member of the caregiver-child dyad will separately complete a standardized questionnaire with pre-worded demographic and pilot-tested survey questions that assess dietary patterns, food security, and food access [20]. The RA will assist children with survey completion. Child body mass index (BMI) percentile and BMI z-score will be calculated based on measured height and weight, as described in the ‘Primary Outcome Measures’ section. Key constructs and study measures are listed in Table 1.

**Table 1:** Key Constructs

Domains	Constructs	Measures	Instruments
**Personal Outcomes**	Child self-efficacy	•Self-efficacy for fruit consumption •Self-efficacy for vegetable consumption •Proxy efficacy to influence parents to make fruits and vegetables available	•Self-efficacy questionnaire [42, 43]
	Caregiver self-efficacy	•Self-efficacy to purchase and consume fruits and vegetables	•Selected questions from Food Attitudes and Behaviors Survey [44]
**Environmental Outcomes**	Exposure to fruit and vegetable prescriptions	•Number of months child has been a patient at fruit and vegetable prescription participating pediatric office •Number of fruit and vegetable prescriptions received	•Medical chart review
	Caregiver dietary patterns	*Mean daily servings of:* •Total fruits •Total vegetables •Total fruits and vegetables	•National Cancer Institute Fruit & Vegetable Intake “All Day” Screener [45, 46]
	Caregiver fruit and vegetable access	•Access to fruits and vegetables •Barriers to access •Social support to access	•Food Attitudes and Behaviors Survey [44] •Michigan Behavioral Risk Factor Surveillance Survey
	Food security	•Perceived food security among youth	•Food Security Survey Module for Youth [47]
		•Household food security and hunger	•US Household Food Security Module: Six-Item Short Form [48]
**Behavioral Outcomes**	Child fruit and vegetable consumption	*Mean daily servings of:* •Total fruits •Total whole fruits •Total vegetables •Total fruits and vegetables	•Two non-consecutive dietary recalls [49] •Block Kids Food Screener [50]
	Child dietary patterns	•Nutrient estimates •Number of servings by food groups •Healthy eating index	•Block Kids Food Screener [50]
**Secondary Outcomes**	Child weight status	•Body mass index (BMI) •BMI percentile, BMI z-score	•Measured weight •Measured height

To compare baseline dietary intake, food security, and weight status between pediatric patients with varying levels of exposure to the FVPP, we will collect study measures at baseline from 700 dyads at each of three partnering clinics. To measure changes in diet, food security, and weight status when never-exposed children are introduced to the FVPP, we will follow approximately 200 dyads from clinic 3, collecting study measures at baseline, 12- and 24-month follow-up. In addition to posters, brochures, and websites that explain the FVPP, caregivers at clinic 3 will be reminded of each follow-up visit through reminder cards and telephone calls.

REDCap provides data security measures designed to prevent unauthorized access, disclosure, alteration, and use of data. Data from the completed evaluation tools will be stored in the REDCap project database. Children and caregivers will be assigned identification numbers upon enrollment in the study. All data will be de-identified within the data management system, and de-identified data will be exported for analyses.

Any modifications to the approved protocol will be submitted to Michigan State University IRB for review and approval prior to implementation. Furthermore, although the likelihood of any adverse event in this trial is remote, we have formalized our Data and Safety Monitoring Plan to include a designated Data and Safety Monitor. This Monitor will have the authority to recommend termination of the trial if he makes the determination that unacceptable adverse events have taken place. Should any adverse events occur, these will be immediately reported to Michigan State University IRB for review.

### Primary outcome measures

#### Child dietary patterns

Because the children’s dietary patterns across gradients of exposure to the FVPP are of primary interest, we will administer two non-consecutive 24-h dietary recalls and one validated food frequency screener. The dietary recall data will cover the 24 h before enrollment and a second 24-h interval that occurs just before a post-baseline assessment completed by the RA within seven days after baseline. The Automated Self-Administered 24-Hour (ASA-24) Dietary Assessment Tool will be used for these assessments. The 24-h dietary recall is a valid measure for assessing the dietary intake of children ages 8 years and older [49]. The RA also will administer Block Kids Food Screener, a food frequency questionnaire, to allow for the assessment of usual and long-term eating behaviors with relatively little administration burden. Block Kids Food Screener has shown good relative validity for children and adolescents [50]. Its 41 items assess the frequency and quantity in which foods and beverages were consumed over the previous week.

After data collection, a dietary analysis, using the Block Online Analysis System, will provide nutrient estimates and the number of servings by food group. We will use these data to evaluate the mean daily intake of total fruits and vegetables, total vegetables, total fruits, and whole fruits. The Healthy Eating Index (HEI) will be generated from the dietary data for use as a measure of the diet quality of the children [51, 52].

#### Child-reported food security

Children ages 12 years and older will complete the Self-Administered Food Security Survey Module for Youth (SAFSSMY). Although the SAFSSMY was found to have adequate internal validity for children ages 12 years and older, it is not recommended for use with younger children [47]. The sum of affirmative responses (“a lot” or “sometimes”) to module questions will provide the child’s initial raw score on the perceived food security scale, with food security status assigned based on raw score (0–1 = high/marginal food security; 2–5 = low food security; 6–9 = very low food security). This study’s sample size is sufficient for a confirmatory factor analysis based on Item Response Theory, which will be used to evaluate whether the unit weight assigned to each item can be improved upon.

#### Household food security

Caregivers will complete the US Household Food Security Module: Six-Item Short Form (National Center for Health Statistics) to assess household food insecurity and hunger [48]. The sum of affirmative responses (“often”, “sometimes”, “yes”, “almost every month”, “some months but not every month”) on the module will serve as the household’s raw score. Food security status will be assigned based on the raw score (0–1 = high/marginal food security; 2–4 = low food security; 5–6 = very low food security). We will use this data to calculate household food security and hunger. Here also, after planned analyses based on the raw score, a confirmatory factor analysis approach will be taken in a post-estimation exploratory analysis.

#### Child weight, height, and BMI status

The trained RA will take two successive measurements of each child’s weight and height within the patient examination room, without shoes or heavy outer garments. Then, the RA will enter the data electronically using the REDCap secure digital platform. Weight will be measured to the closest 0.2 kg on a digital platform scale accurate to 200 kg. Height will be measured to the closest 0.1 cm using a portable stadiometer. Body mass index (BMI), which correlates with more expensive and direct measures of body fat in children (e.g., dual-energy x-ray absorptiometry), will be calculated from child weight and height (weight (kg)/[height (m)]^2^) [53]. The derived BMI can be categorized into normed percentiles by sex and age to serve as an indicator of overweight and obesity [54].

### Secondary outcome measures

#### Self-efficacy

Per our theoretical model, improving self-efficacy for consuming fruits and vegetables is important to change dietary behavior [34]. A validated questionnaire will be administered at each time point to assess caregiver self-efficacy to purchase and consume fruits and vegetables as well as child self-efficacy to consume fruits and vegetables and proxy efficacy to influence parents to make fruits and vegetables available [42, 43].

#### Caregiver dietary patterns

To investigate whether exposure to the FVPP is associated with increased fruit and vegetable consumption among caregivers, dietary data from caregivers will be collected using the National Cancer Institute Fruit & Vegetable Intake “All Day” Screener, which asks frequency and portion size questions about nine food items. This self-administered screener will provide an estimate of the median intake of fruit and vegetable servings in adults [45, 46]. This will be used to calculate the mean daily intake of vegetables and total fruits.

#### Caregiver food access

To evaluate access to fruits and vegetables as well as related barriers and social supports, we will ask caregivers to complete selected questions from the Food Attitudes and Behaviors Survey [44]. Responses will be answered on a 5-point Likert scale, ranging from 1, being “strongly disagree”, to 5, being “strongly agree”. Finally, caregivers will complete four questions related to fruit and vegetable quality and access in neighborhood stores from the Michigan Behavioral Risk Factor Surveillance Survey.

### Covariates

To the extent possible, this study’s potential covariates have been chosen from the NIH Environmental Influences on Child Health Outcomes (ECHO)-wide protocol because these variables are well-established and broadly applicable in dietary research, and each can be assessed using low-burden measurements [55]. Within ECHO, there are many measured variables from the Patient-Reported Outcomes Measurement Information System (PROMIS). PROMIS was developed and validated to assess patient-reported outcomes for clinical research and practice [56].

#### Child physical activity

To assess physical activity, children and adolescents will complete the eight-question PROMIS Physical Activity questionnaire. This instrument will measure a child’s performance of activities that require physical actions.

#### Child sleep patterns

Three measures chosen by the NIH ECHO working group teams will assess sleep-related constructs: PROMIS Sleep Disturbance, PROMIS Sleep-Related Impairment, and ECHO Sleep Ecology. Children will complete these surveys, which were designed for self-administration at ages 8–17 years. Assessing three domains (*n* = 15 total questions) will allow for a more nuanced understanding of how sleep might confound or interact with other variables.

#### Caregiver weight and height

The RA will ask for a self-report of caregiver weight and height. These variables might moderate the estimated associations between FVPP exposure levels and child outcomes.

#### Baseline sociodemographic covariates and other characteristics of interest

This set of baseline covariates, all assessed by the RA, includes household income, caregiver and child education, ethnicity, and age, as well as the name of the neighborhood or community of residence. As noted above, caregiver weight and height (by self-report) also will be included as a covariate and potential moderator. The iPad assessment also will ask the caregiver about participation in food assistance programs (other than the FVPP) and food shopping behaviors.

### Statistical analysis plan

Consistent with general best practices regarding analytic procedures [57], we will employ multiple statistical techniques to test the robustness and sensitivity of our findings across various methods. Specifically, parametric analyses efficient under correct model specification will first be conducted, followed by robust semiparametric and nonparametric analyses. All efforts will also be made during the design and the data collection stages to minimize attrition and the rate of missing data. At the analysis stage, however, multiple imputation techniques coupled with Markov Chain Monte Carlo simulations will be used for handling missing at random mechanisms. Informative missing data mechanisms will be accommodated via pattern-mixture modeling and selection modeling, followed by a sensitivity analysis [58–61].

Because the study calls for a non-randomized design, the analysis will rely on statistical techniques to control for confounders as a source of potential biases. Specifically, a classical approach based on regression adjustment, in which potential confounders are added to the linear predictor, will be adopted. The adjusted analysis will control for well-known confounders between exposure to FVPP and both the primary and secondary outcomes, regardless of their statistical significance. Further comparative analyses will be conducted to determine additional sources of confounders. Specifically, bivariate analyses will be conducted to evaluate the associations between the study group, the study endpoints and the child’s level variables (e.g., age, gender, and race) and the caregiver’s level variables (e.g., household-reported income, education, and other nutrition program involvement).

An alternative balancing strategy based on propensity scores will be used to mitigate potential differences at baseline among participants in the three study groups. These scores will be used to derive an unbiased estimate of the average treatment effect of FVPP on study endpoints. The generalized propensity score to account for multiple levels of treatment in the spirit of Imbens will be estimated with a multinomial logistic regression [62]. To estimate the FVPP effects, we will use an elaborated multinomial logistic model that includes the child’s level, the caregiver’s level covariates, and functions thereof (e.g., interactions).

The choice of analytic methods will be guided by the specific aims, the nature of study endpoints and that of relevant data. Independence models will be used to compare baseline dietary intake, food security, and weight status between pediatric patients with varying levels of exposure to the FVPP. Correlated data models will be used to (1) evaluate changes in diet, food security, and weight status when never-exposed children are introduced to the FVPP; and (2) compare mean follow-up measures of dietary intake, food security, and weight status in the initial no exposure group to baseline measures in the high exposure group.

### Comparison of baseline measures based on FVPP exposure

Multiple linear regression will be used to assess the relationship between mean daily intake of fruits and vegetables and level of exposure to the FVPP. A basic logistic regression model will be used to evaluate the adjusted effect of the child-level exposure to the FVPP on the obesity probability. An ordinal logistic regression model will be used to evaluate the adjusted effects of the child-level exposure to the FVPP on food security (measured as an ordinal outcome). Within this class of models, we will entertain the popular proportional odds model with prior evaluation of the assumption of the proportionality of odds across the ordered levels of food security. These analyses will be adjusted for variables such as food assistance program participation, nutrition education program participation, child race, child age, child gender, caregiver-reported income group, caregiver-reported weight status, caregiver education, and child-reported mean daily intake of fruits and vegetables.

### Evaluation of changes when children are introduced to the FVPP

After baseline data collection, periodic follow-up assessments will be conducted to study changes in diet, food security, and weight status among children newly exposed to the FVPP at clinic 3. Because of the repeated nature of the data generated for this aim, we will consider methods that accommodate the within-child and/or the within-caregiver association. For continuous outcomes (e.g., dietary intake), repeated measures models such as linear mixed models and repeated ANOVA models will be entertained in view of their robustness to certain types of missing data. These classes of models will be used to assess changes from baseline at key points in the study [63, 64]. For ordinal endpoints such as food security (with ordered levels), a generalized linear mixed effects model with cumulative logits will be entertained. To examine whether there is a significant improvement in child-reported food security from baseline, contrast representing the difference in log-odds between baseline and follow-up will be evaluated. Alternatively, GEE counterparts of these likelihood-based models (that use the sandwich-based correction in lieu of the model-based variance–covariance matrix of the parameter estimates) will also be considered [65].

### Comparison of mean follow-up measures in the initial no exposure group to baseline measures in the high exposure group

At follow-up, measures of dietary intake, food security, and weight status in a group of children newly exposed to the FVPP will be compared to baseline measures of children with high exposure to the FVPP (> 24 months). For example, to compare measures of dietary intake (at 12 months and 24 months) in the newly exposed group to those of the highly exposed group, the class of linear mixed models and repeated ANOVA models will be considered. These models will be similar to those of aim 2, with the additional flexibility of group-specific time-point effects. For ordinal outcomes (e.g., food security) and binary outcomes (e.g., obesity), a generalized linear mixed effects model will be used. Alternative models such as GEE models will also be entertained.

## Discussion

Although the current US Farm Bill provides funding opportunities for produce prescription programs, there are multiple knowledge gaps related to duration and intensity of exposure; ideal program timing and length; implementation components; and overall effectiveness. Current FVPPs vary widely in content, scope, duration, and intensity across the US, although most programs target adults with diet-related chronic health conditions and offer fruit and vegetable prescriptions as a disease-management strategy [22, 23]. The current study is unique in design as it emphasizes the critical role fruits and vegetables play in disease prevention using a pediatric FVPP that has previously demonstrated feasibility [26]. Furthermore, rich and productive partnerships with a network of pediatricians and agricultural leaders across Flint and Michigan have been created. These partnerships, alongside consistent feedback from caregivers and families [28], have aided in the development of this reproducible program that considers fundamental challenges with FVPP implementation within busy clinical settings.

Authors recognize that prescription redemption rates will certainly play a key role in the success of the program. Pilot studies have demonstrated an increase in redemption rates over time, as caregivers and children become more familiar with the FVPP. Additionally, authors have developed brochures, websites, and social media platforms with prescription redemption sites, hours, and instructions that will be available to families at each partnering clinic. Although not a study aim, prescription redemption rates, as well as barriers to redemption of prescriptions, will be monitored and recorded throughout the study period to improve FVPP utilization.

A Randomized Controlled Trial (RCT) was considered as an alternative to the current research study. However, physicians and community partners questioned whether a placebo-controlled RCT would be ethical when the children in Flint face hunger and food insecurity. Hence, we begin with this quasi-experiment, and if successful, we plan for an adaptive approach. For example, in a future RCT, the prescription dollar value (size) might be jittered (e.g., ± $6) around a mean of $15, to elicit estimates based on a dose–response approach. This study will guide us toward that future alternative step in this line of research. Additionally, our previous work has supported the idea that pediatrician messaging, which is easy to implement and replicate, alongside one $15 fruit and vegetable prescription will ensure high prescription distribution. However, should results of this study indicate a need to shift in the direction of adaptive interventions that include a greater focus on educational and behavioral support components, we will adjust the FVPP accordingly.

Completion of study aims will provide evidence for the effectiveness of FVPPs and offer insights regarding the duration and intensity of exposure necessary to influence changes in food security, dietary patterns, and weight status when fruit and vegetable prescriptions are provided to pediatric patients. Study results will be shared broadly at local, state, and national conferences as well as through peer-reviewed publications. Findings will be particularly important to pediatricians and primary care physicians seeking tangible solutions to address both food insecurity and poor dietary habits among young patients. Furthermore, the results of this study are likely to inform policy decisions related to state and federally supported nutrition prescription programs.

## Data Availability

The final de-identified dataset will be shared with a National Institutes of Health-supported data repository. This dataset will be removed of all identifiers. Information will be shared under a strict data-sharing agreement that provides for: (1) a commitment to using data only for research purposes and not to identify any individual participant; (2) a commitment to securing the data using appropriate computer technology; and (3) a commitment to destroying or returning the data after analyses are completed.

## Abbreviations

FVPP: Fruit and vegetable prescription program
SCT: Social Cognitive Theory
EMR: Electronic medical record
FFM: Flint Farmers’ Market
eCW: EClinicalWorks
RA: Research assistant
REDCap: Research Electronic Data Capture
BMI: Body mass index
ASA-24: Automated Self-Administered 24-Hour
HEI: Healthy Eating Index
SAFSSMY: Self-Administered Food Security Survey Module for Youth
ECHO: Environmental Influences on Child Health Outcomes
PROMIS: Patient-Reported Outcomes Measurement Information System
RCT: Randomized controlled trial
